# Supplementary material for: MetaRibo-Seq measures translation in microbiomes
Source: Nat Commun. 2020 Jun 29;11:3268. doi: 10.1038/s41467-020-17081-z (PMC7324362; doi:10.1038/s41467-020-17081-z)
Supplement: Supplementary file 10 — Supplementary Data 7 [file 41467_2020_17081_MOESM10_ESM.zip › File2/Confidence_High_Taxonomy/407357_out.krona.html]

Javascript must be enabled to view this page.

members
magnitude
magnitudeUnassigned
count
unassigned
taxon
rank

407357\_out

2

2
2
superkingdom

phylum
976
2

class
200643
2

order
171549
2

2
171552
family

genus
838
2


SRS077641\_contig\_number\_5844
species
1
2292054

165179
1
species

SRS011134\_contig\_number\_7359
